# Determinants of outcome of solitary fibrous tumors of the pleura: an observational cohort study

**DOI:** 10.1186/1471-2466-14-138

**Published:** 2014-08-12

**Authors:** Daniel Franzen, Matthias Diebold, Alex Soltermann, Didier Schneiter, Peter Kestenholz, Rolf Stahel, Walter Weder, Malcolm Kohler

**Affiliations:** 1 Division of Pulmonology, University Hospital Zurich, Rämistrasse 100, 8091 Zurich, Switzerland; 2 Institute for Surgical Pathology, University Hospital Zurich, Rämistrasse 100, 8091 Zurich, Switzerland; 3 Division of Thoracic Surgery, University Hospital Zurich, Rämistrasse 100, 8091 Zurich, Switzerland; 4 Division of Medical Oncology, University Hospital Zurich, Rämistrasse 100, 8091 Zurich, Switzerland

**Keywords:** Solitary fibrous tumor, Pleura, Immunohistochemistry, Outcome, Proliferation rate

## Abstract

**Background:**

Solitary fibrous tumors of the pleura (SFTP) are rare and their long-term outcome is difficult to predict, as there are insufficient data which allow accurate characterization of the malignant variant. Thus the aim of this study was to describe the outcome and possible determinants of malignant behavior of SFTPs.

**Methods:**

Data were collected retrospectively from medical records of patients treated at the University Hospital Zurich from 1992 to 2012. Kaplan-Meier and Cox regression analysis were performed to define disease-free survival time (defined as survival without tumor-recurrence or tumor-related death) using the classical histo-morphological criteria (tumor size, localization, pedunculation, tumor necrosis or hemorrhage, mitotic activity and nuclear pleomorphism) and immunohistochemical parameters.

**Results:**

42 patients (20 males) with SFTP (median (IQR) age 62 (56–71) years) could be identified. SFTP were associated with symptoms in 50% of all cases. Complete resection was achieved by video-assisted thoracic surgery or thoracotomy in 20 and 22 patients, respectively. Three SFTP-related deaths (7.1%) and four tumor recurrences (9.5%) were observed. Mean disease-free survival time was 136.2 (±13.1) months, and 2-, 5- and 10-year disease-free survival was 91%, 84%, and 67%, respectively. Mean disease-free survival inversely correlated with the mean tumor diameter, number of mitotic figures and proliferation rate (Ki-67 expression). Other criteria (tumor necrosis, atypical localization, sessile tumor, and pleomorphism) were not statistically significant prognostic parameters.

**Conclusions:**

Patients with large SFTP with a high mitotic index and high proliferation rate should be followed-up closely and over a prolonged time period in order to recognize recurrence of the SFTP early and at a treatable stage. Future research on this topic should focus on the prognostic role of immunohistochemistry including Ki-67 expression and molecular parameters.

## Background

Solitary fibrous tumors of the pleura (SFTP) account for less than 5% of all pleural tumors and have a reported incidence of 2.8 cases/100’000 persons per year [1,2]. Since their first description in 1931, approximately 1000 cases have been described in the literature. Historically, the taxonomy of these tumors was heterogeneous. The terms included “localized mesothelioma” or “submesothelial fibroma” reflecting the disagreement concerning the etiology and pathogenesis of these neoplasms [3]. More recently, SFTP were shown to arise from the submesothelial layer, as expression of vimentin and CD34 - both markers of mesenchymal cells - was detected [4,5]. The majority of these tumors follow a benign course with a high cure rate [2,6]. However, features of malignancy are found in 7-60% [6-8], and in such tumors a high recurrence rate and excess mortality have been reported [9]. In addition, some benign SFTP may transform into the malignant variant, sometimes several years after complete surgical resection [10].

To date, there is insufficient information to predict the biological behavior of these tumors which is partly related to very few data on immunohistochemical and molecular markers and limited long-term follow-up data in SFTP [11-13]. Hence, there is a need to define parameters that characterize the malignant variant based on clinico-pathological, immunohistochemical, or molecular findings. The objective of this study was to describe the clinicopathological presentation of SFTP and its impact on the outcome.

## Methods

### Subjects and data

Between January 1, 1992 and December 31, 2012, all consecutive patients with histologically proven SFTP treated at the University Hospital Zurich were included in the present study. Data on demographics, the initial presentation and surgical treatment were collected from medical records. Follow-up data were collected from routine postoperative surveillance examinations, which were performed every six months for five years and yearly thereafter with low-dose computed tomography, or from contact with general practitioners. Written informed consent was obtained from all patients or their relatives. The study was approved by the Ethics committee of the Canton of Zurich, Switzerland (KEK-ZH 2012–0279).

### Outcomes und definition of malignancy

The primary variable of interest of the study was disease-free survival time. Events were defined either as tumor-related death, recurrence of the tumor or evidence of metastases. Malignant SFTP were defined according to the historical criteria proposed by Okike et al. [2] if one or more of the following features were present: (1) high cellularity, (2) high mitotic activity with more than four mitotic figures per 10 high-power fields (HPF), and (3) pleomorphism.

### Possible predictors of outcome

In addition to the aforementioned three pathologic features [2], possible predictors of outcome were clinicopathologic factors described by England et al. [6]: (a) tumor size greater than 10 cm in diameter, (b) atypical localization (SFTP attached to parietal pleura, fissure, or mediastinum, or inverted into lung parenchyma), (c) sessile or pedunculated tumor, (d) existence of necrosis or hemorrhage, (e) more than four mitoses per 10 high-power fields (HPF), and (f) nuclear pleomorphism (expressed as increased nuclear grades). Furthermore, demographic (age, gender, smoking status), clinical (symptoms, radiological findings), and immunohistochemical variables (Ki-67, Cytokeratin, Desmin, Vimentin, CD 34, CD 99, bcl-2 (B-cell lymphoma 2 protein), SMA (smooths muscle antigen), and S-100) were investigated for their possible predictive value on disease-free survival. Ki-67 is a nuclear protein that is expressed in proliferating cells, and thus a surrogate marker of the cellular proliferation rate. It is immunostained with MIB-1 according to standard protocols (DAKO M7240, 1:20) using the Ventana automated Benchmark staining system (MIB-1 labeling index).

### Statistical analysis

All statistical analyses were performed using IBM SPSS Statistics for Windows, version 20.0 (IBM Corporation, Armonk, NY). Data are reported as median ± interquartile range (IQR), or mean ± standard deviation (SD), or percentages as appropriate. Differences between malignant and benign SFTP were assessed using χ^2^ tests. Kaplan-Meier analysis was used to estimate disease-free survival. Univariate analysis by Cox regression analysis was performed to evaluate the possible predictive value on disease-free survival of the above mentioned covariates. Variables with a *p*-value of less than 0.05 in the univariate analysis were entered into the multiple regression model. For statistical significant variables, the hazard ratio (HR) with a 95% confidence interval (CI) was calculated. *P*-values of all outcomes were two-sided; a value less than 0.05 was considered to indicate statistical significance.

## Results and discussion

### Demographic and clinical data

Between 1992 and 2012, a total of 42 patients (20 males) with histologically proven SFTP were treated at the University Hospital Zurich. Demographic, clinical and radiological data are summarized in Table 1.

**Table 1:** Preoperative demographic, clinical and radiological characteristics of SFTP

**Characteristics**		**N = 42**
Demographics	Male gender, N (%)	20 (47.6)
	Age [years]	63.0 (55.7-71.0)
	Current or history of smoking, N (%)	22 (52.4)
	Pack years of smoking (py)	2.5 (0–21.2)
Symptoms	No symptoms	21 (50.0)
	Dyspnea	11 (26.2)
	Cough	10 (23.8)
	Chest pain	10 (23.8)
	Constitutional symptoms (night sweats, weight loss)	6 (14.3)
	Digital clubbing	4 (9.5)
	Symptomatic hypoglycemia	2 (4.7)
Laboratory results	Hemoglobin [g/l]	137.0 (117.0-149.0)
	Leucocytes count [× 10^9^/l]	7.2 (5.8-8.7)
	C-reactive protein [mg/l]	3.0 (2.0-14.0)
	Blood glucose [mmol/l]	5.4 (5.1-6.3)
Radiology	Extra-pulmonary mass	37 (88.1)
	Inhomogeneous mass	17 (40.5)
	Contact to pleura	37 (88.1)
	Pleural effusion	5 (11.9)
	Displacing other anatomical structures	13 (31.0)
	Invasion into other anatomical structures	2 (4.8)
	Focal calcification	5 (11.9)
	Localization in lower lobes	22 (52.4)

Values are median (IQR) where applicable.

### Therapy and pathology

SFTP’s were resected by a wedge resection (75.6%) followed by lobectomy (17.1%) or pneumonectomy (7.3%) in cases where the tumor was extensive and adherent to the lung. In 20 patients (47.6%), the operation was performed by thoracoscopy. R0-resection (tumor-free margins) was achieved in all patients irrespective of the surgical method. Perioperative complications were seen in seven patients (16.7%) including prolonged air leak (n = 3), infection (n = 2), bleeding (n = 1), and intercostal neuralgia (n = 1). The mean length of hospital stay was 6 (IQR 4–9) days, and perioperative mortality was 0%.

Eighteen SFTP (42.9%) were classified as malignant. The histomorphological and immunohistochemical profile of SFTP are summarized in Table 2.

**Table 2:** Histomorphological and immunohistochemical profile of SFTP

	**All (n = 42)**	**Malignant (n = 18)**	**Benign (n = 24)**	*p*-value*
**Histomorphology**				
Atypical localization	2 (4.8)	1 (5.6)	1 (4.2)	ns
Tumor size [cm]	6.2 (4.8-14.1)			
Tumor size > 10 cm	19 (45.2)	12 (66.7)	7 (29.2)	0.002
Sessile tumor	11 (26.2)	7 (38.9)	4 (16.7)	ns
Necrosis	19	11 (61.1)	7 (29.2)	0.024
Nr of mitoses per 10 HPF	4.1 (1.0-7.25)			
> 10 mitoses per 10 HPF	14 (33.3)	13 (72.2)	0 (0)	0.022
Pleomorphism	8 (19.0)	6 (33.3)	2 (8.3)	0.03
**Immunohistochemistry**				
Ki-67 (proliferation rate)**	5 (1–7)	13.3	3.2	0.01
Ki-67 > 12%**	3/27 (11.1)	3 (30)	0	0.02
Vimentin	16/18 (88.9)	8 (88.9)	8 (88.9)	ns
Cytokeratin	1/19 (5.3)	0 (0)	1 (10.0)	ns
Desmin	4/16 (25.0)	3 (33.3)	1 (14.3)	ns
SMA	1/18 (5.6)	1 (12.5)	0 (0)	ns
CD 34	35/36 (97.2)	16 (94.1)	19 (100)	ns
CD 99	14/18 (77.8)	5 (55.6)	9 (100.0)	ns
bcl-2	21/22 (95.5)	8 (88.9)	13 (100.0)	ns
S-100	0/23 (0)	0 (0)	0 (0)	ns

Values are N (%) or median (IQR) where applicable.

*Chi-quare test; ns, not significant; HPF, high-power field; SMA, smooth muscle antigen; bcl-2, B-cell lymphoma 2 protein; **Proliferations rate is determined by Ki-67 expression (MIB-1 labeling index).

### Disease-free survival

**Figure 1:**
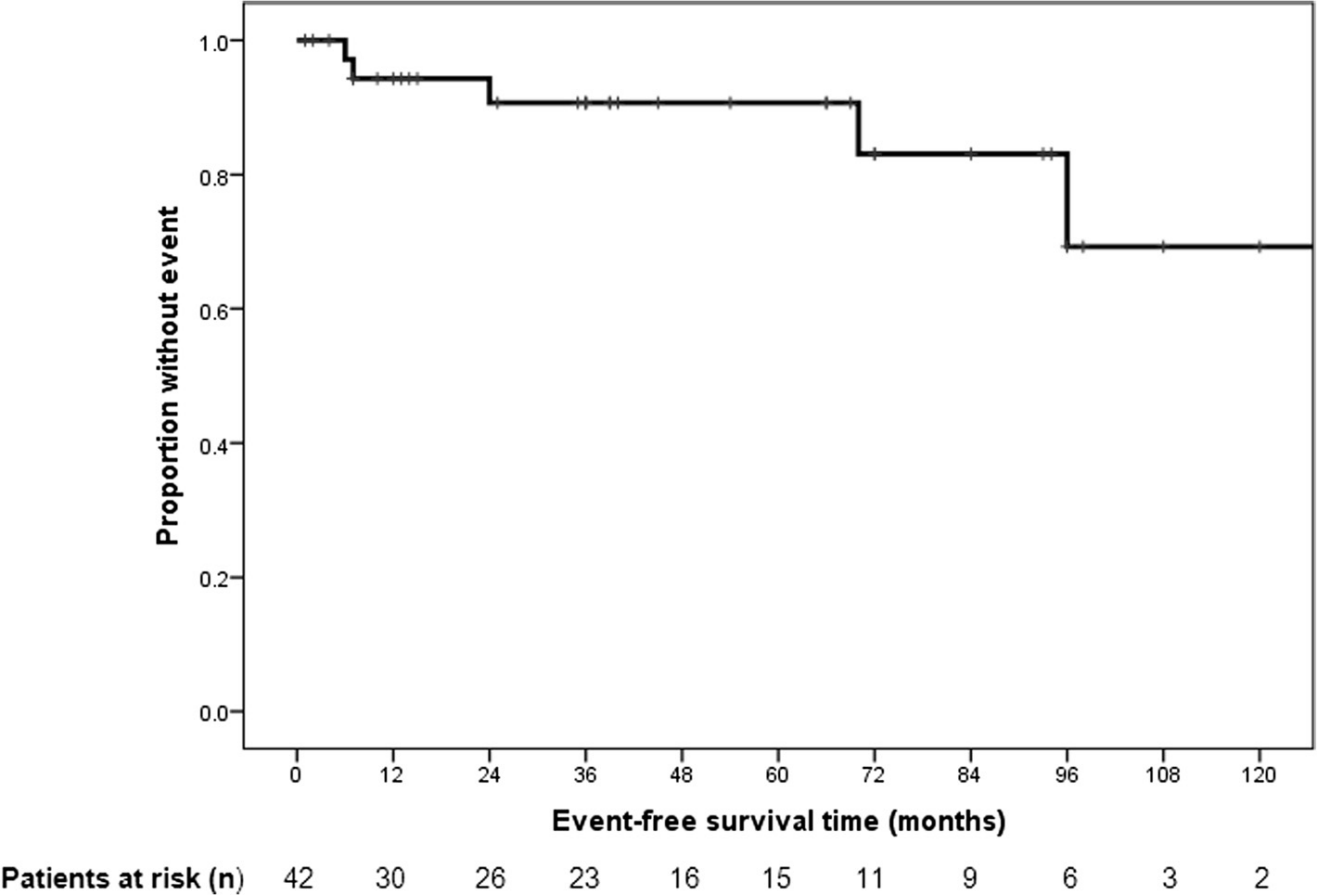
**Disease-free survival in solitary fibrous tumors of the pleura.** Mean disease-free survival was 136.2 (±13.1) months, and 2-, 5- and 10-year disease-free survival was 91%, 84%, and 67%, respectively.

Mean disease-free survival was 136.2 (±13.1) months, and 2-, 5- and 10-year disease-free survival was 91%, 84%, and 67%, respectively (Figure 1). During the median follow-up time of 39 (14–78) months, the endpoint (recurrence or SFTP-related death) was reached in five patients (11.9%). Recurrence of SFTP was found in four patients (9.5%), and SFTP-related mortality was 7.1%.

### Univariate analysis

**Figure 2:**
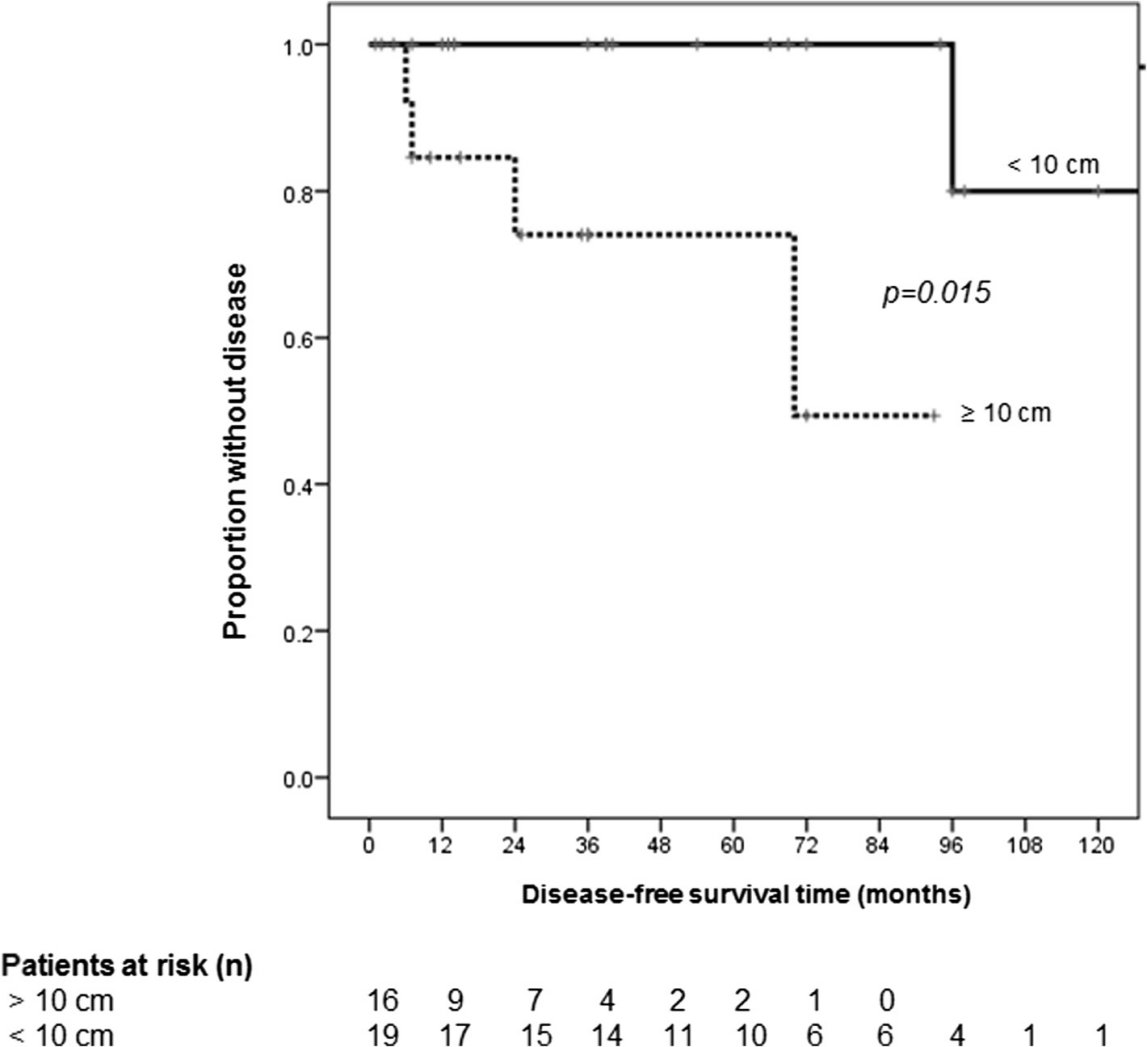
**Disease-free survival grouped by tumor diameter.** Mean disease-free survival time in solitary fibrous tumors of the pleura with tumor diameter greater than 10 cm was 66.7 (±10.4) compared to 153.6 (±12.9) months in smaller tumors.

**Figure 3:**
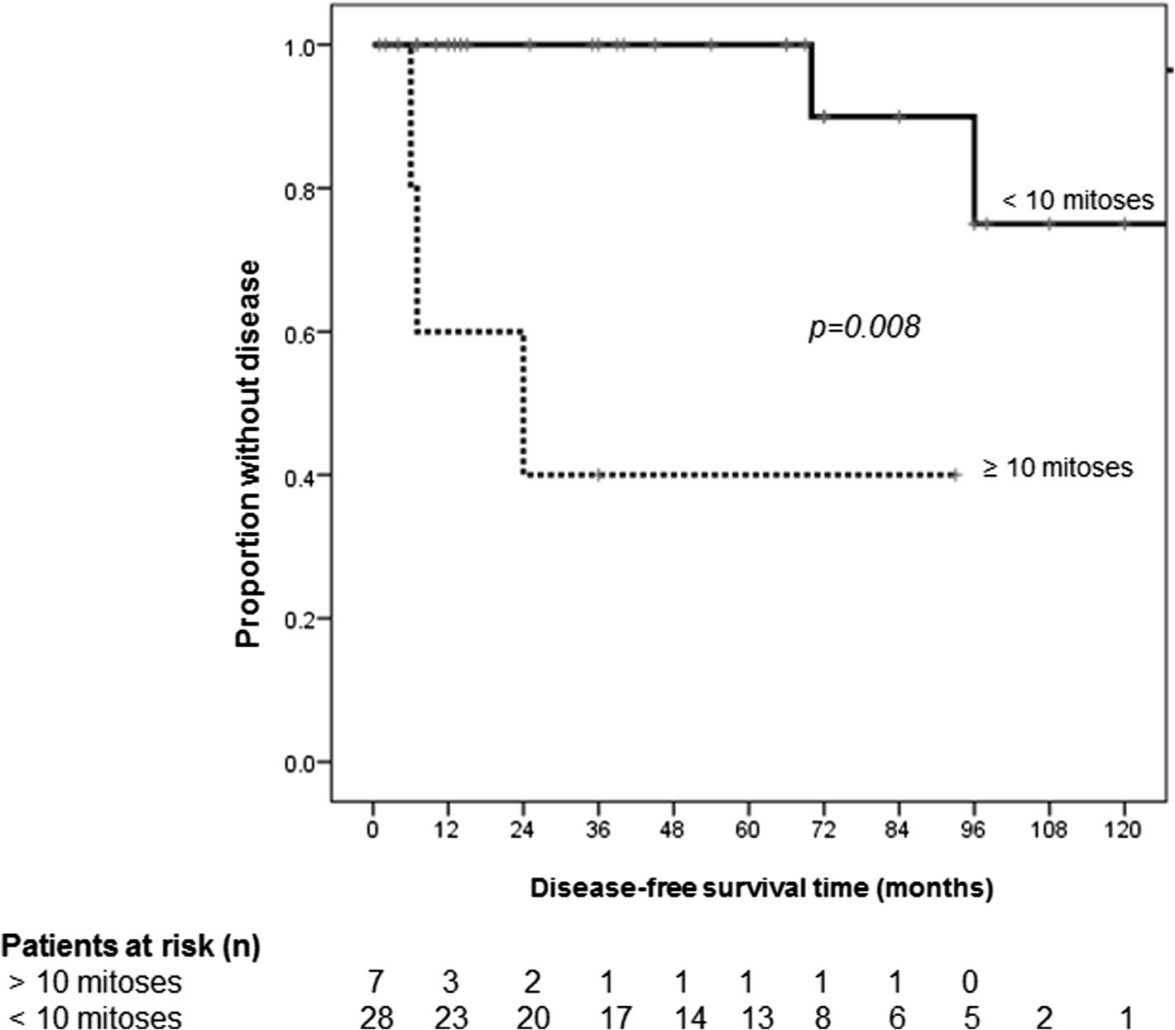
**Disease-free survival grouped by number of mitotic figures.** Mean disease-free survival time in solitary fibrous tumors of the pleura with a number of mitotic figures over 10 per 10 high-power fields was 44.6 (±17.9) compared to 147.4 (±12.8) months in tumors with less mitoses.

**Figure 4:**
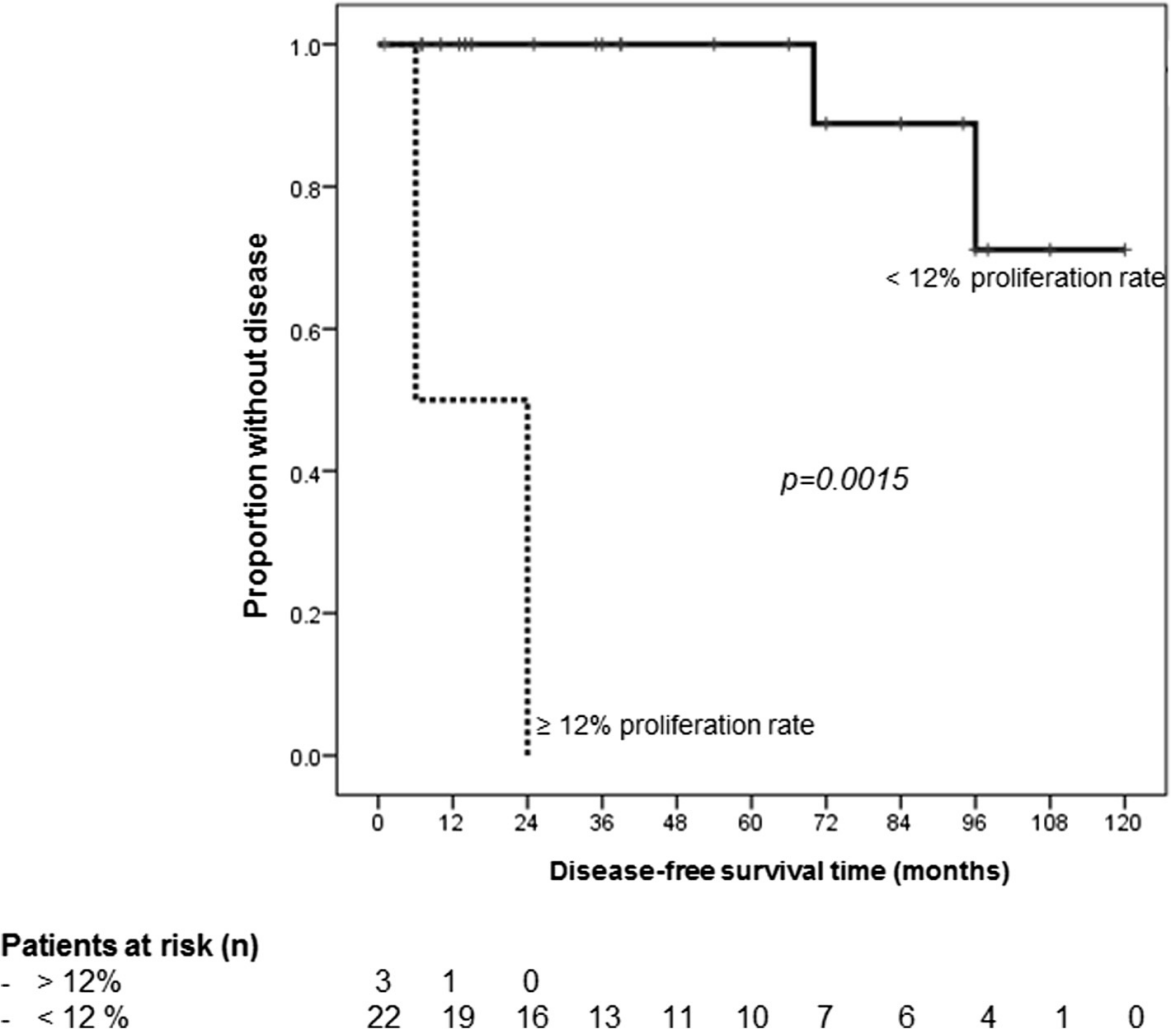
**Disease-free survival grouped by proliferation rate (Ki-67).** Mean disease-free survival time in solitary fibrous tumors of the pleura with a proliferation rate (Ki-67 expression, MIB-1 labeling index) greater than 12% was 15.0 (±9.0) compared to 110.2 (±6.1) months in those with less proliferation rate.

In univariate Cox regression analysis, large tumor size (HR 1.24, 95% CI 1.04-1.47, p = 0.015), high mitotic rate (HR 1.33, 95% CI 1.08-1.63, p = 0.008), and high proliferation rate (HR 1.09, 95% CI 1.02-1.16, p = 0.015) were significantly associated with impaired disease-free survival (Table 3). All patients with high proliferation rate also had a mitotic rate over 10/HPF. Comparative disease-free survival curves in SFTP with and without tumor diameter greater than 10 cm, number of mitotic figures over 10 per 10 HPF, and proliferation rate (Ki-67, MIB-1) greater than 12% are displayed in Figures 2, 3 and 4. Hence, from the clinicopathologic features proposed by England et al. [6], only tumor size and the number of mitoses showed a statistically significant association with disease-free survival, whereas atypical localization, sessile or pedunculated tumor, existence of necrosis or hemorrhage, and pleomorphism were not predictive for disease-free survival. Furthermore, the classification in malignant and benign variants according to the pathologic features published by Okike et al. [2] showed no statistically significant association with disease-free survival (Table 3).

**Table 3:** Investigated variables with a possible impact on the outcome of patients with solitary fibrous tumors of the pleura

**Variable**	**HR**	**CI**	*p*-value*
**Univariate analysis**			
Gender	0.54	0.89-3.26	ns
Age	1.13	0.91-1.30	ns
History of smoking	2.26	0.29-2.35	ns
Symptomatic SFTP	1.19	0.20-7.14	ns
Inhomogeneous appearance on CT	4.69	0.48-45.52	ns
Sessile SFTP	3.90	0.43-35.04	ns
Necrosis or hemorrhage	0.14	0.02-1.31	ns
Atypical localization	0.04	0.03-4.3	ns
Malignant SFTP according to Okike classification [2]	0.21	0.02-2.00	ns
Pleomorphism	0.45	0.07-2.74	ns
Desmin	2.35	0.43-5.34	ns
Vimentin	3.65	0.33-40.32	ns
CD 34	1.0	0.8-2.2	ns
CD 99	4.84	0.44-53.81	ns
Bcl-2	5.32	0.87-7.34	ns
SMA	2.29	0.03-8.54	ns
Tumor diameter ≥ 10 cm	1.24	1.04-1.47	0.015**
≥ 10 mitotic figures/10 HPF	1.33	1.08-1.63	0.008**
Proliferation rate ≥ 12%***	1.09	1.02-1.16	0.015**
**Multivariate analysis**			
Tumor diameter ≥ 10 cm	12.45	0.28-5578.61	ns
≥ 10 mitotic figures/10 HPF	2.29	0.29-18.12	ns
Proliferation rate ≥ 12%***	1.53	0.35-6.64	ns

*Cox regression analysis; **Variables with a p-value ≤ 0.05 were entered into the multivariate analysis; ***Determined by Ki-67 expression (MIB-1 labeling index); HR, hazard ratio; CI, confidence interval; CT, computed tomography; ns, not significant; HPF, high-power field, SFTP, solitary fibrous tumor of the pleura; SMA, smooth muscle antigen; bcl-2, B-cell lymphoma 2 protein.

### Multivariate analysis

In multiple regression analysis, none of the features, which were significantly associated with disease-free survival in the univariate analysis, remained statistically significantly associated with disease-free survival after correction for covariates.

### Discussion

In univariate analysis, impaired outcome in patients with histologically proven SFTP inversely correlated with mean tumor diameter, number of mitotic figures per 10 high-power fields, and proliferation rate (measured with Ki-67, a nuclear protein that is expressed in proliferating cells and immunostained with MIB-1). However, these associations did not remain significant in the multivariate analysis, possibly due to the limited number of included cases. Beyond complete resection of SFTP, which has been shown to be a major prognostic indicator by several authors [2,6,14-16], and was achieved in all our cases, several histomorphologic, immunohistochemical, or molecular features may correlate with prognosis. However, the evidence concerning factors predicting a worse outcome is conflicting throughout the literature. Histomorphologic characteristics apparently predicting aggressive behavior (including local recurrence, metastases or SFTP-related death) were first described by England et al. [6] and included tumor size greater than 10 cm in diameter, atypical localization, sessile tumor, existence of necrosis or hemorrhage, more than four mitoses per 10 high-power fields (HPF), and nuclear pleomorphism. However, none of these factors seems to accurately predict an impaired outcome, as 59% of SFTP may display at least one of the above mentioned features, whereas mortality and recurrence have only been observed in 10% and 18% of all cases, respectively [11].

More recently, de Perrot et al. proposed a staging system mainly based on the sessile morphology of SFTP and on the above mentioned characteristics in order to identify SFTP at high risk of aggressive behavior [17]. In 2008, this modified staging system was evaluated by Schirosi et al. [11] who were able to show a significant correlation with overall and disease-free survival. However, in the multivariate analysis of the latter study, the authors found that only tumor necrosis and high p53 expression were parameters significantly associated with overall and disease-free survival [11].

So far only a few immunohistochemical and molecular parameters with a possible impact on prognosis in SFTP have been investigated. High p53 expression has previously been shown to correlate with malignancy by others [18,19]. Also, loss of CD34 expression and cytokeratin (CK) positivity was considered to correlate with unfavorable outcome in SFTP [18,20-22]. Recently, the prognostic value of CD34 negativity was confirmed in a large retrospective study conducted in 157 patients with SFTP [23]. The same authors confirmed that sessile morphology was a significant risk factor for recurrence, but interestingly tumor size was not associated with the outcome [23]. Other previously mentioned histomorphological features were not considered in the latter study [23]. Unexpectedly the authors found no significant difference of survival between patients with malignant and those with benign SFTP, whereas recurrence was more common in patients with malignant variants [23].

In contrast to some previous studies [6,17,23], we did not find that sessile configuration of SFTP was significantly associated with the outcome. Similarly, Lococo et al. did not find a significant association between sessile morphology and outcome in SFTP [16].

In the current study, prognosis was not affected by patient’s age, the site (visceral or parietal pleura) or by the existence of pleural effusion. Also, in univariate analysis, characterization as malignant or benign tumors according to the criteria by Okike et al. [2] was not significantly predictive for disease-free survival. From the clinicopathologic features described by England et al. [6], only tumor size and mitotic rate were significantly correlated with the outcome, which confirms previous findings [6,12,17]. In the current study, high proliferation rate, assessed by Ki-67, a nuclear protein that is expressed in proliferating cells, and immunostained with MIB-1, significantly correlated with adverse outcome. Ki-67 expression was previously considered as prognostic parameter in other studies including relatively small series consisting of up to 18 subjects [24-26]. However, despite markers of an increased cell proliferation have been shown to correlate with worse prognosis in patients with other tumors, such as breast [27,28] and bladder cancer [29], the role of Ki-67 immunostaining has not been systematically investigated in SFTP so far. Recently, a cut off level of a proliferation rate of 12% (Ki-67) was proposed to indicate benign and malignant lesions [30]. However, assessment of Ki-67 may suffer from inter- and intra-observer variability, since there is an area of uncertainty whether and how MIB-1 labeling index should be counted or estimated [31].

Regarding the role of other immunohistochemical staining methods for predicting prognosis, we could not confirm the findings of others concerning CD34 negativity [18,23]. In our study, we were not able to identify any immunohistochemical marker other than Ki-67 (including positivity for Vimentin, CD99, or bcl-2, and negativity for CD34, SMA, and S-100) to correlate with disease-free survival. However, the role of immunohistochemical (particularly Ki-67 expression) and distinct molecular techniques (including p16 expression) have yet to be determined in a future study. Furthermore, the prognostic role of rearrangement of chromosome 8 according to some case reports [32,33] and the role of microRNAs has to be investigated.

The present study has some inherent limitations mainly due to the retrospective design. The sample size in our study is relatively small, which might impair the interpretability of the multivariate analysis and the overall-survival analysis. For this reason we have chosen to display the disease-free survival only. However, we feel that the findings of our study merit attention, because proliferation rate in SFTP has been considered as a possible prognostic parameter in very few and small case series only. Noteworthy, our series is the largest investigating the proliferation rate measured with Ki-67 immunostaining and its impact on outcome in SFTP.

## Conclusions

Tumor-related mortality, recurrence or metastases are difficult to predict in SFTP solely based on demographic, histo-pathological or clinical parameters. High mitotic rate, large tumor size, and high proliferation rate seem to be associated with adverse outcome. Hence, to date the definition of malignant SFTP should refer to these parameters.

## Competing interests

The authors declare that they have no competing interests.

## Authors’ contribution

Study design (DF, AS, MK), data collection (DF, AS, MD, DS, PK, WW, MK), data analysis (DF, MD AS, MK), drafting of the manuscript (DF, RS, MK). Approval of the final version of the manuscript (all authors).

## Pre-publication history

The pre-publication history for this paper can be accessed here:

http://www.biomedcentral.com/1471-2466/14/138/prepub

## References

[B1] Lu C Ji Y Shan F Guo W Ding J Ge D Solitary fibrous tumor of the pleura: an analysis of 13 cases World J Surg 2008 32 8 1663 1668 10.1007/s00268-008-9604-y 18427887

[B2] Okike N Bernatz PE Woolner LB Localized mesothelioma of the pleura: benign and malignant variants J Thorac Cardiovasc Surg 1978 75 3 363 372 633933

[B3] Cardillo G Lococo F Carleo F Martelli M Solitary fibrous tumors of the pleura Curr Opin Pulm Med 2012 18 4 339 346 10.1097/MCP.0b013e328352f696 22450304

[B4] Al-Izzi M Thurlow NP Corrin B Pleural mesothelioma of connective tissue type, localized fibrous tumour of the pleura, and reactive submesothelial hyperplasia: an immunohistochemical comparison J Pathol 1989 158 1 41 44 10.1002/path.1711580109 2754539

[B5] Flint A Weiss SW CD-34 and keratin expression distinguishes solitary fibrous tumor (fibrous mesothelioma) of pleura from desmoplastic mesothelioma Hum Pathol 1995 26 4 428 431 10.1016/0046-8177(95)90145-0 7535740

[B6] England DM Hochholzer L McCarthy MJ Localized benign and malignant fibrous tumors of the pleura: a clinicopathologic review of 223 cases Am J Surg Pathol 1989 13 8 640 658 10.1097/00000478-198908000-00003 2665534

[B7] Cardillo G Facciolo F Cavazzana AO Capece G Gasparri R Martelli M Localized (solitary) fibrous tumors of the pleura: an analysis of 55 patients Ann Thorac Surg 2000 70 6 1808 1812 10.1016/S0003-4975(00)01908-1 11156076

[B8] Suter M Gebhard S Boumghar M Peloponisios N Genton CY Localized fibrous tumours of the pleura: 15 new cases and review of the literature Eur J Cardiothorac Surg 1998 14 5 453 459 10.1016/S1010-7940(98)00213-9 9860200

[B9] Robinson LA Solitary fibrous tumor of the pleura Cancer Control 2006 13 4 264 269 17075563 10.1177/107327480601300403

[B10] de Perrot M Kurt AM Robert JH Borisch B Spiliopoulos A Clinical behavior of solitary fibrous tumors of the pleura Ann Thorac Surg 1999 67 5 1456 1459 10.1016/S0003-4975(99)00260-X 10355431

[B11] Schirosi L Lantuejoul S Cavazza A Murer B Yves Brichon P Migaldi M Sartori G Sgambato A Rossi G Pleuro-pulmonary solitary fibrous tumors: a clinicopathologic, immunohistochemical, and molecular study of 88 cases confirming the prognostic value of de Perrot staging system and p53 expression, and evaluating the role of c-kit, BRAF, PDGFRs (alpha/beta), c-met, and EGFR Am J Surg Pathol 2008 32 11 1627 1642 10.1097/PAS.0b013e31817a8a89 18753943

[B12] Demicco EG Park MS Araujo DM Fox PS Bassett RL Pollock RE Lazar AJ Wang WL Solitary fibrous tumor: a clinicopathological study of 110 cases and proposed risk assessment model Mod Pathol 2012 25 9 1298 1306 10.1038/modpathol.2012.83 22575866

[B13] Kohler M Clarenbach CF Kestenholz P Kurrer M Steinert HC Russi EW Weder W Diagnosis, treatment and long-term outcome of solitary fibrous tumours of the pleura Eur J Cardiothorac Surg 2007 32 3 403 408 10.1016/j.ejcts.2007.05.027 17646108

[B14] Briselli M Mark EJ Dickersin GR Solitary fibrous tumors of the pleura: eight new cases and review of 360 cases in the literature Cancer 1981 47 11 2678 2689 10.1002/1097-0142(19810601)47:11<2678::AID-CNCR2820471126>3.0.CO;2-9 7260861

[B15] Rosado-de-Christenson ML Abbott GF McAdams HP Franks TJ Galvin JR From the archives of the AFIP: localized fibrous tumor of the pleura Radiographics 2003 23 3 759 783 10.1148/rg.233025165 12740474

[B16] Lococo F Cesario A Cardillo G Filosso P Galetta D Carbone L Oliaro A Spaggiari L Cusumano G Margaritora S Graziano P Granone P Malignant solitary fibrous tumors of the pleura: retrospective review of a multicenter series J Thorac Oncol 2012 7 11 1698 1706 10.1097/JTO.0b013e3182653d64 23070244

[B17] de Perrot M Fischer S Brundler MA Sekine Y Keshavjee S Solitary fibrous tumors of the pleura Ann Thorac Surg 2002 74 1 285 293 10.1016/S0003-4975(01)03374-4 12118790

[B18] Yokoi T Tsuzuki T Yatabe Y Suzuki M Kurumaya H Koshikawa T Kuhara H Kuroda M Nakamura N Nakatani Y Kakudo K Solitary fibrous tumour: significance of p53 and CD34 immunoreactivity in its malignant transformation Histopathology 1998 32 5 423 432 10.1046/j.1365-2559.1998.00412.x 9639117

[B19] Morimitsu Y Nakajima M Hisaoka M Hashimoto H Extrapleural solitary fibrous tumor: clinicopathologic study of 17 cases and molecular analysis of the p53 pathway APMIS 2000 108 9 617 625 10.1034/j.1600-0463.2000.d01-105.x 11110050

[B20] Yan B Raju GC Salto-Tellez M Epithelioid, cytokeratin expressing malignant solitary fibrous tumour of the pleura Pathology 2008 40 1 98 99 10.1080/00313020701716417 18038330

[B21] Sanchez-Mora N Cebollero-Presmanes M Monroy V Carretero-Albinana L Herranz-Aladro M Alvarez-Fernandez E Clinicopathological features of solitary fibrous tumors of the pleura: a case series and literature review Arch Bronconeumol 2006 42 2 96 99 10.1157/13084401 16539940

[B22] Cavazza A Rossi G Agostini L Roncella S Ferro P Fedeli F Cytokeratin-positive malignant solitary fibrous tumour of the pleura: an unusual pitfall in the diagnosis of pleural spindle cell neoplasms Histopathology 2003 43 6 606 608 10.1111/j.1365-2559.2003.01703.x 14636263

[B23] Lahon B Mercier O Fadel E Ghigna MR Petkova B Mussot S Fabre D Le Chevalier T Dartevelle P Solitary fibrous tumor of the pleura: outcomes of 157 complete resections in a single center Ann Thorac Surg 2012 94 2 394 400 10.1016/j.athoracsur.2012.04.028 22704328

[B24] Sung SH Chang JW Kim J Lee KS Han J Park SI Solitary fibrous tumors of the pleura: surgical outcome and clinical course Ann Thorac Surg 2005 79 1 303 307 10.1016/j.athoracsur.2004.07.013 15620963

[B25] Hanau CA Miettinen M Solitary fibrous tumor: histological and immunohistochemical spectrum of benign and malignant variants presenting at different sites Hum Pathol 1995 26 4 440 449 10.1016/0046-8177(95)90147-7 7705824

[B26] Hiraoka K Morikawa T Ohbuchi T Katoh H Solitary fibrous tumors of the pleura: clinicopathological and immunohistochemical examination Interact Cardiovasc Thorac Surg 2003 2 1 61 64 10.1016/S1569-9293(02)00091-9 17669989

[B27] Colozza M Azambuja E Cardoso F Sotiriou C Larsimont D Piccart MJ Proliferative markers as prognostic and predictive tools in early breast cancer: where are we now? Ann Oncol 2005 16 11 1723 1739 10.1093/annonc/mdi352 15980158

[B28] Reyal F Hajage D Savignoni A Feron JG Bollet MA Kirova Y Fourquet A Pierga JY Cottu P Dieras V Fourchotte V Laki F Alran S Asselain B Vincent-Salomon A Sigal-Zafrani B Sastre-Garau X Long-term prognostic performance of Ki67 rate in early stage, pT1-pT2, pN0, invasive breast carcinoma PLoS One 2013 8 3 e55901 10.1371/journal.pone.0055901 23526930 PMC3602517

[B29] Stavropoulos NE Filiadis I Ioachim E Hastazeris K Tsimaris I Kalogeras D Stefanaki S Agnantis NJ Prognostic significance of p53, bcl-2 and Ki-67 in high risk superficial bladder cancer Anticancer Res 2002 22 6B 3759 3764 12552989

[B30] Moghaddam NA Rahmani A Taheri D Desfuli MM Proliferative index using Ki-67 index in reactive mesothelial versus metastatic adenocarcinoma cells in serous fluid Adv Biomed Res 2012 1 29 10.4103/2277-9175.98155 23210088 PMC3507028

[B31] Varga Z Diebold J Dommann-Scherrer C Frick H Kaup D Noske A Obermann E Ohlschlegel C Padberg B Rakozy C Sancho Oliver S Schobinger-Clement S Schreiber-Facklam H Singer G Tapia C Wagner U Mastropasqua MG Viale G Lehr HA How reliable is Ki-67 immunohistochemistry in grade 2 breast carcinomas? A QA study of the Swiss working group of breast- and gynecopathologists PLoS One 2012 7 5 e37379 10.1371/journal.pone.0037379 22662150 PMC3360682

[B32] de Leval L Defraigne JO Hermans G Dome F Boniver J Herens C Malignant solitary fibrous tumor of the pleura: report of a case with cytogenetic analysis Virchows Arch 2003 442 4 388 392 12715174 10.1007/s00428-002-0754-2

[B33] Horton ES Dobin SM Donner LR A clonal t(8;12)(p11.2;q24.3) as the sole abnormality in a solitary fibrous tumor of the pleura Cancer Genet Cytogenet 2007 172 1 77 79 10.1016/j.cancergencyto.2006.07.015 17175385

